# Tumor suppressor genes promote rhabdomyosarcoma progression in p53 heterozygous, HER-2/neu transgenic mice

**DOI:** 10.18632/oncotarget.1171

**Published:** 2013-08-06

**Authors:** Marianna L. Ianzano, Stefania Croci, Giordano Nicoletti, Arianna Palladini, Lorena Landuzzi, Valentina Grosso, Dario Ranieri, Massimiliano Dall'Ora, Ilaria Santeramo, Milena Urbini, Carla De Giovanni, Pier-Luigi Lollini, Patrizia Nanni

**Affiliations:** ^1^ Department of Experimental, Diagnostic and Specialty Medicine, University of Bologna.; ^2^ Laboratory of Experimental Oncology, “Rizzoli” Orthopedic Institute, Bologna, Italy.; ^3^ Present address: Clinical Immunology, Allergy and Advanced Bio-technologies Unit, IRCCS – Arcispedale Santa Maria Nuova, Reggio Emilia, Italy.

**Keywords:** rhabdomyosarcoma, p53, HER-2, p19Arf, p21Cip1

## Abstract

Human sarcomas arise suddenly, thus preempting the study of preneoplastic and early neoplastic lesions. To explore the natural history of these tumors we studied male mice carrying a heterozygous deletion of *p53* and an activated *HER-2/neu* transgene (BALB-p53Neu mice), that develop urethral rhabdomyosarcomas with nearly full penetrance and early onset (4 months of age). Among genes prominently upregulated in preneoplastic tissue, and more highly expressed in tumors, we found the insulin-like growth factor 2 (*Igf2*) and tumor suppressors, *p19Arf* and *p21Cip1*. In urethral tissues of male mice *p53* was less expressed than in female mice, whereas *HER-2/neu* was more expressed, a combination not found in other skeletal muscles of the same mice that could contribute to the anatomic and sexual specificity of BALB-p53Neu rhabdomyosarcoma. Upregulation of *p19Arf* and *p21Cip1* was additively determined by *HER-2/neu* activation and by *p53* inactivation. Silencing of *p19Arf* or *p21Cip1* in rhabdomyosarcoma cell lines can inhibit cell growth and motility, thus suggesting that these genes can contribute to growth autonomy and malignancy of tumor cells. *In vivo* injection of gene-silenced cells highlighted selective variations in organ-specific metastatic ability, indicating that overexpression of *p19Arf* and *p21Cip1* controlled both tumor cell-intrinsic properties and microenvironmental interactions. The onset of pelvic rhabdomyosarcoma in BALB-p53Neu male mice is triggered by the coincidental overexpression of *HER-2/neu* and hypoexpression of the residual *p53* allele, that foster *p53* loss, Igf2 autocriny and overexpression of *p19Arf* and *p21Cip1*, a phenotype that could provide novel potential targets for cancer prevention and therapy.

## INTRODUCTION

Alterations of tumor suppressor genes play a prominent role in cancer development, alone or in combination with activated oncogenes. From a therapeutic perspective, inactivated tumor suppressor genes are problematic targets, especially when inactivation results from the lack of a tumor suppressor protein, rather than from a mutant with dominant negative activity.

The loss of a pleiotropic tumor suppressor gene, like *p53*, may promote carcinogenesis through direct and indirect mechanisms. In some cancers overexpression of tumor suppressor proteins was demonstrated [1–3], possibly resulting from compensatory mechanisms or from the interruption of homeostatic feed-back circuits. In turn, overexpression of tumor suppressors might be either an irrelevant, “passenger” phenomenon, or a driving event in the genesis of specific tumor types. In the latter case, such “reactive”, hyperexpressed tumor suppressor genes might provide new therapeutic targets, to develop an anti-compensatory therapy to inhibit cancer progression.

We have developed a mouse model of rhabdomyosarcoma that combines one inactivated allele of the tumor suppressor *p53* and one activated allele of the *HER-2/neu* oncogene [4]. All male mice develop rhabdomyosarcoma in the genitourinary tract around 4 months of age, with remarkable gender specificity (only males, not females, are affected by rhabdomyosarcoma), anatomic specificity (only urethral striated muscle proximal to the urinary bladder gives rise to tumors) and genetic specificity (urethral rhabdomyosarcoma only affects bigenic mice, but not parental mice carrying either a *p53* inactivated allele or a *HER-2/neu* mutant allele).

Human rhabdomyosarcomas, and sarcomas in general, arise abruptly, thus preempting the study of molecular events leading to tumor development. The repeatable and predictable spontaneous carcinogenesis of our mouse model allowed us to investigate early events of rhabdomyosarcomagenesis. To our surprise, here we found that some tumor suppressor genes were specifically upregulated, in particular cyclin-dependent kinase inhibitor 1A (*CDKN1A/p21Cip1*) and cyclin-dependent kinase inhibitor 2A (*CDKN2A/p19Arf*).

p21Cip1 belongs to the Cip/Kip family of cyclin-dependent kinases (CDK) inhibitors. It is one of the mediators of p53 activities and its promoter contains two p53-responsive elements [5]. Knockout mice develop histiocytic sarcomas, hemangiomas, and lymphomas with a mean latency of 16 months [6], thus revealing a role of p21Cip1 in the control of tumor development.

The *CDKN2A* locus encodes two different proteins: p16Ink4a and p19Arf (p14Arf in humans), transcribed from two different promoters. They share exon 2 and exon 3 while differ in exon 1. p19Arf is translated in an alternative reading frame (Arf), thus it differs from the p16Ink4a protein both regarding aminoacid sequence and biological function [7]. The best characterized function of p19Arf is the activation of *p53*, that in turn induces the expression of cell cycle inhibitory genes and apoptosis inductors. p19Arf blocks the ubiquitin ligases Mdm2 and Arf-BP1/Mule (Arf-binding protein1/Mcl1-ubiquitin ligase E3) thus decreasing ubiquitination, nuclear export and degradation of p53. Recently it has been found that p19Arf can also have p53-independent functions. It can inhibit ribosome biogenesis by binding nucleophosmin and can induce sumoylation of its binding proteins. Finally, a short mitochondrial form of p19Arf (smArf) can modify mitochondrial membrane potential and promote autophagy [7]. Knockout mice lacking the entire *CDKN2A* locus [8], *p19Arf* alone [3], or *p16Ink4a*alone [9] develop mainly sarcomas and lymphomas [10]. On the whole, current studies indicate that *p21Cip1* and *CDKN2A* are tumor suppressors with a proven role either upstream or downstream of *p53*.

We show here that overexpression of p21Cip1 and p19Arf during rhabdomyosarcoma development in a *p53*-defective system can enhance clonogenicity, motility and metastatic capacity of rhabdomyosarcoma cells.

## RESULTS

### Gene expression in preneoplastic and neoplastic BALB-p53Neu mice

Urethral rhabdomyosarcoma of male *HER-2/neu*-transgenic, *p53^+/−^* mice (BALB-p53Neu mice) develops around four months of age. To identify genes potentially involved in the genesis of rhabdomyosarcoma, gene expression was studied in preneoplastic (8 weeks of age) urethral tissue and was compared with that of mice not prone to rhabdomyosarcoma, *i.e.* parental and BALB/c mice. We used PCR arrays (see Methods section) to study 420 genes in five pathways known to be relevant for muscle development or to be deregulated in rhabdomyosarcoma. Twenty genes showed a >2-fold change expression in the preneoplastic urethra of male BALB-p53Neu mice, and even higher levels of expression were found in tumors, than in urethral tissues of BALB/c mice (Table 1), thus suggesting an early involvement in the genesis of rhabdomyosarcoma.

**Table 1:** Genes up-regulated in the preneoplastic urethral tissue of 8-week-old and in rhabdomyosarcomas (RMS) of male BALB-p53Neu mice relative to the urethral tissues of male BALB/c

Gene symbol	Description	Urethral thissue of BALB-p53Neu vs BALB/c	RMS vs urethral tissue of BALB/c
Cdkn2a	Cyclin-dependent kinase inhibitor 2a	200	1490
IGF2	Insulin-like growth factor 2	147	1446
MyoD1	Myogenic differentiation 1	36	180
Pmaip1	Phorbol-12-myristate-13-acetate induced protein 1	25	139
Cdkn1a	Cyclin-dependent kinase inhibitor 1a (p21)	17	49
Nog	Noggin	12	23
Bdnf	Brain-derived neurotrophic factor	9	65
Cdon	Cell adhesion molecule-related/down-regulated by oncogenes	8	31
Nes	Nestin	8	62
Cdc2a	Cell division cycle 2 homolog A (S. pombe)	6	18
Cdkn2b	Cyclin-dependent kinase inhibitor 2b (p15)	6	94
Grb10	Growth factor receptor bound protein 10	5	23
Ccnb2	Cyclin B2	4	42
Serpine1	Serine (or cysteine) peptidase inhibitor, clade E, member 1	4	8
Cdc25c	Cell division cycle 25 homolog c (S. pombe)	3	19
Birc5	Baculoviral IAP repeat-containing 5	3	17
Vcan	Versican	3	12
Wnt3	Wingless-related MMTV integration site 3	3	9
Igfbp3	Insulin-like growth factor binding protein 3	2	20
Gdf6	Growth differentiation factor 6	2	17

Figures rep resent the fold change relative to the indicated comparison (see Methods for calculations)

Insulin-like growth factor 2 (*Igf2*) was highly expressed among the twenty genes that were precociously expressed in preneoplastic tissues. Igf2 is known to drive an autocrine circuit in human and mouse rhabdomyosarcoma [4, 11], hence its overexpression prior to tumor development supported the validity of this screening approach. Less expected was the up-regulation of *p21Cip1* and of *p19Arf*, therefore we decided to further investigate their possible involvement in the onset of urethral rhabdomyosarcoma in male BALB-p53Neu mice.

### Preneoplastic gene expression

To define genetic and sexual determinants in the genesis of rhabdomyosarcoma, we compared gene expression of primary tumors and of preneoplastic urethral tissue of BALB-p53Neu mice with urethral tissues of various congenic mice not prone to rhabdomyosarcoma onset, including female BALB-p53Neu mice, “wild-type” BALB/c mice (indicated as *p53^+/+^* for clarity) and mice carrying either inactivated *p53* alleles (both *p53^+/−^* and *p53^™/™^*), or an activated *HER-2/neu* transgene.

**Figure 1:**
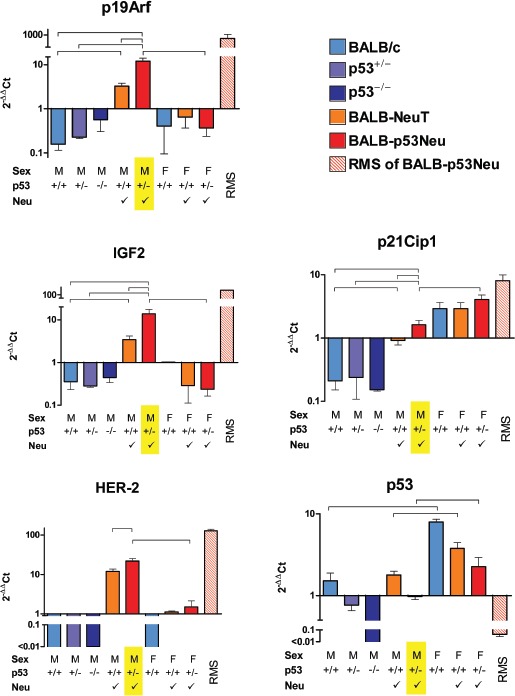
Gene expression in urethral tissues of 8-week-old mice and in rhabdomyosarcomas (RMS) of BALB-p53Neu mice Each panel represents the expression of the indicated gene in samples obtained from mice differing in sex (M/F), *p53* status *(p53^+/+^, p53^+^* or and presence of a HER-2/neu transgene *(S)* as indicated under each bar; predisposition to RMS is highlighted in yellow. Each bar in a panel represents expression level relative to the average of all samples (excluding RMS). Mean threshold cycles were: *GAPDH*, 20.1; *p19Arf*, 32.4; *Igf2*, 26.8; *p21Cip1*, 24.7; *HER-2/neu*, 33.1; *p53*, 25.5. The number of mice in each group was: BALB/c male mice (M +/+) 4; *p53* heterozygous BALB/c males (M +/™>) 3; *p53* knockout males (M ™/™) 2; male BALB-NeuT (M +/+ *S*) 10; male BALB-p53Neu (M +/™ *S*) 7; female BALB/c (F +/+) 3; female BALB-NeuT (F +/+ *S*) 5; female BALB-p53Neu (F +/™*S\* 5; rhabdomyosarcomas (RMS) of male BALB-p53Neu, 2. Horizontal lines above bars indicate selected significant (p<0.05 at least) statistical comparisons by the Student's *t* test.

In summary, male mice bearing both *HER-2* and *p53* gene mutations showed *p19Arf*, *Igf-2* and *p21Cip1* expression levels significantly higher than those observed in wild-type mice or in mice bearing single mutations (Figure 1). No significant difference relative to parental strains (either *p53^+/−^* or *HER-2/neu* trangenic mice) was found in a different striated muscle, the quadriceps, which is not prone to rhabdomyosarcoma development (Supplementary Figure 1). Therefore the differential expressions observed in urethral tissues were peculiar of the site of tumor onset.

*p19Arf* and *Igf-2* were specifically up-regulated in primary rhabdomyosarcomas and in preneoplastic urethral tissue of BALB-p53Neu male mice in comparison to BALB-NeuT and urethral tissue of wild-type male mice (Figure 1), whereas they were significantly down-regulated in urethral tissue of female mice compared to male mice (Figure 1). Therefore these early alterations might be involved in the gender- and strain-specific rhabdomyosarcoma genesis.

*p19Arf* and *Igf-2* showed a higher expression in urethral tissue of male *HER-2/neu* transgenic than in wild-type mice (Figure 1), thus indicating that activated *HER-2/neu* could predispose the high expression of the two genes. No difference of expression was found between *p53^+/−^* and *p53^+/+^* male mice (Figure 1). *p19Arf*, but not *Igf-2*, was also increased in *p53^™/™^* male mice (Figure 1). Therefore the high expression of *p19Arf* in preneoplastic tissues, already evident in non-rhabdomyosarcoma prone urethral tissues of *HER-2/neu* transgenic mice and of *p53^™/™^* mice, was further increased by the combined action of *HER-2/neu* activation and *p53* inactivation. *Igf-2* was modulated only by *HER-2/neu* activation, but in BALB-p53Neu mice the combination of *HER-2/neu* activation and *p53* inactivation produced a higher *Igf2* expression than in all other mice (Figure 1).

*p21Cip1* was overexpressed in primary rhabdomyosarcomas and in preneoplastic male urethral tissue, as compared to *HER-2/neu* transgenic male mice, however it was also expressed at high level in female mice, which are not prone to rhabdomyosarcoma development, furthermore it was not up-regulated in the absence of *p53* (Figure 1). In conclusion, the pattern of overexpression of *p21Cip1* indicated that the activation of the pathway leading to p21Cip1 increase could be insuffcient for the onset of rhabdomyosarcoma.

### Tissue- and sex-specific gene expression

Anatomical specificity of rhabdomyosarcoma development, as opposed to random tumor onset in any striated muscle, is a distinctive property not only of BALB-p53Neu mice, but also of other rhabdomyosarcoma-prone, genetically-modified mouse models [11]. Therefore it was important to determine the expression of *p53* and *HER-2/neu* during tumor development, in particular because it has not been previously measured at the site of rhabdomyosarcoma development.

*HER-2/neu* was significantly more expressed in urethral tissue of BALB-p53Neu male mice than in *HER-2/neu* transgenic mice, and it was more expressed in rhabdomyosarcomas and in urethral tissue of males than in females (Figure 1). In general *p53* expression was directly proportional to the number of functional alleles. However we found a sex-specific variation in *p53^+/+^* and in *p53^+/−^* mice, that showed lower *p53* levels in urethral tissues of male mice relative to females (Figure 1). The profiles of *HER-2/neu* and *p53* transcripts suggested their involvement in gender-related rhabdomyo sarcoma genesis in BALB-p53Neu mice: in the urethral tissue, male mice expressed *HER-2/neu* about 15 times more than female mice, and expressed *p53* about two times less than female mice (Figure 1). Therefore *p53*-heterozygous male mice could be more exposed to tissue-specific genomic instability than females, thus leading to the development of *p53*-negative urethral rhabdomyosarcoma (Figure 1 and [4]).

No significant difference between males and females in the expression of *HER-2/neu* and *p53* was found in a muscle immune from rhabdomyosarcoma development (Supplementary Figure 1).

### Silencing of p21Cip1 and CDKN2A inhibited growth and motility of rhabdomyosarcoma cells

**Figure 2:**
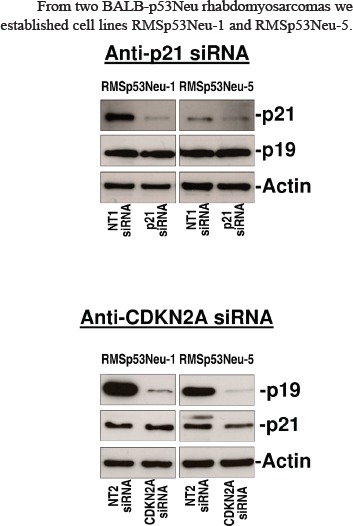
Gene silencing in rhabdomyosarcoma cell lines with siRNAs against *p21Cip1* or *CDKN2A* specifically inhibited target gene expression Western blot was performed as reported in Methods section.

From two BALB-p53Neu rhabdomyosarcomas we established cell lines RMSp53Neu-1 and RMSp53Neu-5. Both cell lines expressed high protein levels of p21Cip1 and p19Arf. Treatment with anti-*p21Cip1* siRNA strongly and specifically inhibited the expression of p21Cip1 (Figure 2). Silencing of p21Cip1 did not affect the expression of p19Arf (Figure 2). Protein expression of p19Arf was strongly inhibited by anti-*CDKN2A* siRNAs (Figure 2). The expression of p21Cip1 was not affected by *CDKN2A* silencing (Figure 2).

**Figure 3:**
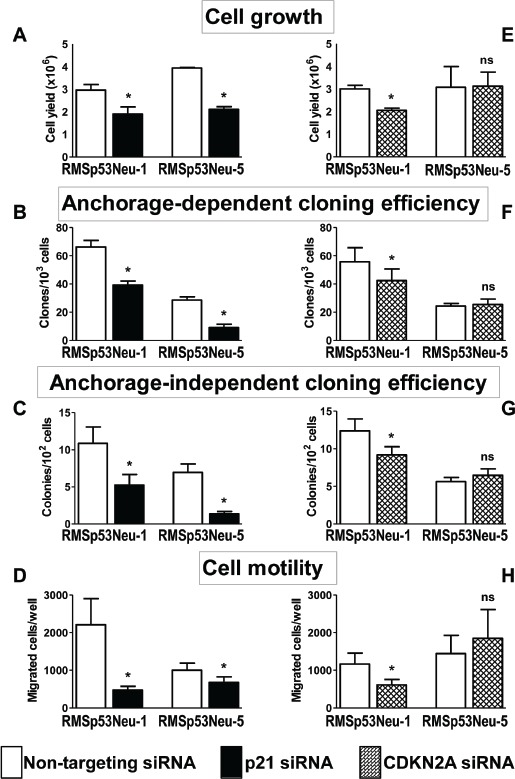
Silencing of p21Cip1 (*A-D*) or CDKN2A (*E-H*) inhibits growth, clonogenicity and motility of rhabdomyosarcoma cells See Methods for methodological details. Each bar in a panel represents the mean ± SEM of 3-9 replicates. Stars over bars indicate significant (p<0.05, at least) statistical comparisons by the paired Student's *t* test *versus* cells treated with Non-targeting siRNAs.

Treatment of rhabdomyosarcoma cell lines with anti-*p21Cip1* siRNA significantly inhibited cell growth (Figure 3A). Cloning efficiency was specifically inhibited by siRNA treatment both under anchorage-dependent (Figure 3B) and anchorage-independent conditions (Figure 3C). Silencing of p21Cip1 also inhibited the migratory ability of both rhabdomyosarcoma cell lines (Figure 3D), with a stronger effect on RMSp53Neu-1 (80% in comparison to cells treated with control siRNA) than on RMSp53Neu-5 (30%).

Silencing of CDKN2A in RMSp53Neu-1 cells resulted in a specific inhibition of cell growth, cloning efficiency and cell motility, on the contrary, the growth, cloning efficiency and motility of RMSp53Neu-5 cells were not inhibited (Figure 3 E-H).

### Inhibition of metastasis by p21Cip1 and CDKN2A silencing

Cell migration and clonal growth are among those properties that determine the metastatic ability of malignant tumors. The inhibitory effects of siRNA shown above prompted us to study the metastatic potential of rhabdomyosarcoma cells after *in vitro* silencing of p21Cip1 or of CDKN2A.

Silencing of p21Cip1 reduced lung metastases by both RMSp53Neu-1 and RMSp53Neu-5 cells (Table 2), but did not affect liver metastases. Treatment with anti-*CDKN2A* siRNA, similar to what happened *in vitro (*see above), produced differential results on the two cell lines. Silenced RMSp53Neu-1 cells showed decreased lung and liver colonization ability, whereas RMSp53Neu-5 cells resulted less metastatic in the liver site, but more metastatic in the lung (Table 2).

**Table 2:** Inhibition of metastatic ability by silencing of p21 or CD KN2A

Cell line#	Metastatic site	siRNA treatment	Metastasis		
			Incidence	Median	Range
					
RMSp53Neu1	Lung	Non-targeting 1	6/7	11	0-15
		Anti-p21	4/7	1**	0-4
RMSp53Neu5	Lung	Non-targeting 1	5/5	121	73-194
		Anti-p21	5/5	7**	2-19
					
RMSp53Neu1	Liver	Non-targeting 1	1/7	0	0-1
		Anti-p21	0/7	0	0-0
RMSp53Neu5	Liver	Non-targeting 1	5/5	26	7-52
		Anti-p21	5/5	25	17-38
					
RMSp53Neu1#	Lung	Non-targeting 2	9/9	26	12-51
		Anti-CDKN2A	9/9	7**	1-26
RMSp53Neu5	Lung	Non-targeting 2	5/5	60	12-122
		Anti-CDKN2A	5/5	157*	112-201
					
RMSp53Neu1#	Liver	Non-targeting 2	9/9	2	1-5
		Anti-CDKN2A	3/9	0**	0-1
RMSp53Neu5	Liver	Non-targeting 2	5/5	52	7-81
		Anti-CDKN2A	4/5	9*	0-29

# Cells treated for 48 h with siRNAs were injected i.v. in male BALB-NeuT mice. RMSp53Neu 1 treated with Non-Targeting 2 or anti-CDKN2A siRNAs were injected in Rag2−/−;Il2rg−/− mice (see Methods).

* p<0.05

** p<0.01 by the Student's t test versus corresponding Non-targeting siRNA.

## DISCUSSION

The natural history of human tumors that, like rhabdomyosarcoma and many other sarcomas, arise suddenly, is largely unknown. Current knowledge of tumor progression from normal cell to malignant neoplasm is mostly based on epithelial cancers that have a long natural history, punctuated by successive preneoplastic and early neoplastic featuring distinct molecular abnormalities relative to the surrounding normal tissue [12].

The unpredictable onset of most human non-epithelial tumors prevented a direct analysis of preneoplastic events, but the development of genetically-modified animal models, prone to the development of specific tumor types and with a defined natural history, now offers unique opportunities not only to investigate repeatable sequences of oncogenic events, but also to generate hypotheses that can be subsequently tested in human cancer, eventually leading to the definition of novel targets for cancer therapy.

The study of preneoplastic tissue of BALB-p53Neu mice reported here revealed a high expression of *p19Arf* and *p21Cip1*, thus suggesting that these genes could have an oncogenic role in rhabdomyosarcoma development. Gene silencing demonstrated a functional role of these genes in sustaining growth and malignancy of rhabdomyosarcoma, thus indicating novel potential therapeutic targets.

The oncogenic, pro-tumor activities shown here contrast with known tumor suppressor functions of these genes. A major role in this phenomenon could be played by the *p53* status of cells, in fact tumor-suppressing functions of these genes have been mainly defined in *p53*-profcient cells, whereas their activities in *p53*-defcient cells are more heterogeneous. Furthermore, the cell and tissue contexts of gene expression, as well as the specific genes activated or inactivated during the carcinogenic processes, determine the balance between pro- and anti-oncogenic activities [13, 14]. In our system it was evident that the inactivation of *p53* and the activation of *HER-2/neu* acted in an additive manner in inducing the observed increase of p19Arf and p21Cip1.

p19Arf is overexpressed in various human tumors, in particular when *p53* is mutated [15]. This might be an irrelevant byproduct of *p53* loss, however recent evidence demonstrates that p19Arf overexpression can have relevant activities that favor tumor growth and progression [16]. In murine lymphoma cells exposed to metabolic stress, p19Arf was found to induce autophagy in a p53-independent manner, and to protect cells from nutrient deprivation, eventually favoring tumor growth *in vivo* [15]. In *PTEN* knockout mice p19Arf expression fostered the development of prostate cancer, whereas *p19Arf* deletion partially inhibited carcinogenesis [17]. Analogous conclusions were reached concerning the role of p19Arf in the development of sarcomas induced by *K-ras* [18]. In summary, in addition to its known tumor-suppressing activity, in specific contexts, and in particular in the absence of *p53*, p19Arf was found to favor carcinogenesis, exactly as it appears to do in our rhabdomyosarcoma model. It remains to be determined whether in this case p19Arf exerts its oncogenic activity through the activation of autophagy [16].

We found that metastatic colonization of different organs was differentially affected by *CDKN2A* siRNAs, thus suggesting that both cell-intrinsic properties and interactions with systemic circulation or organ microenvironment were involved. The different metastatic behavior of RMSp53Neu-1 and RMSp53Neu-5 after CDKN2A silencing might refect tumor heterogeneity, and suggest the need of further studies to test the role of CDKN2A as a therapeutic target. These results, together with data on human tumors cited above, compose an intriguing picture and indicate various directions for further investigations. In particular we plan to study the *Rb* pathway during rhabdomyosarcoma development and to dissect the reciprocal role of p19Arf, p16Ink4a and p15Ink4b using selective gene silencing. We also plan to investigate the relation between *CDKN2A* gene overexpression and tissue-specific invasion and metastatic growth.

A further kinase inhibitor, p21Cip1, participated in the development and progression of BALB-p53Neu rhabdomyosarcoma. Overexpression of p21Cip1 in human sarcomas was shown several years ago [19], followed by studies on the progression of carcinomas [20, 21]. Studies in genetically-modifed mice confirmed a possible oncogenic role [22], in particular in the absence of *p53* [23]. The mechanisms proposed to explain oncogenic activity included inhibition of apoptosis and transcriptional activation of secreted growth and survival factors [24]. A decade of studies expanded the range of molecular mechanisms, that now include also the promotion of cyclin D activity and the definition of differential p21Cip1 functions depending on subcellular localization [14, 25, 26]. Cytoplasmic p21Cip1 induced by HER-2/neu [27] can promote cell growth and motility [28]. As in previous cases, this data suggests several perspectives in the study of RMSp53Neu rhabdomyosarcoma development, in particular the subcellular localization of the p21Cip1 protein in our cells.

A further general question to which the BALB-p53Neu model could contribute is that of the anatomical specificity of carcinogenesis. In most human cancer syndromes only a very specific set of tissues and organs is affected by carcinogenesis, even though the altered genes are ubiquitously expressed. This is also the case of different mouse models of rhabdomyosarcoma that combine *p53* inactivation with a second genetic alteration. In our case the activation of *HER-2/neu* produced rhabdomyosarcomas exclusively in the proximal urethra; *Fos* knockout lead to periorbital tumors [29]; *Ras* activation to tumors predominantly on the limbs [30]. The studies reported here revealed significant tissue-specific differences in the expression of *p53* and *HER-2/neu* that could portend the pattern of tumor development. We found that the preneoplastic proximal urethra of male BALB-p53Neu mice expressed more *HER-2/neu* and less *p53* than that of females, and that such differential expression was not present in skeletal muscles not affected by rhabdomyosarcoma development. Sex- and tissue-specific differences in the expression of the *HER-2/neu* transgene (controlled by a mouse mammary tumor virus long terminal repeat) are known to occur and to affect carcinogenesis [31]. To the best of our knowledge, the reduced expression of *p53* in specific, tumor-prone tissues of male mice is reported here for the first time, and could synergize with the increased expression of *HER-2/neu* in the same tissue in determining the onset of rhabdomyosarcoma. Higher level of *p53* in urethral tissue of female mice could be due to the activity of estrogens as an upmodulation of p53 protein levels has been reported in cells treated with estradiol [32].

Finally, are the results obtained in the BALB-p53Neu model system relevant for human rhabdomyosarcoma? This is tantamount to asking whether rhabdomyosarcomas molecularly and pathologically similar to those of BALB-p53Neu exist in human pathology. Deregulation of the p53 pathway is prevalent in human rhabdomyosarcoma, resulting from direct mutation of the *p53* gene and/or by alterations of up/downstream genes [11]. A well known growth factor circuit implicated in the genesis of human rhabdomyosarcoma is based on coexpression of IGF2 and its receptor IGF1R [11], a feature shared also by the rhabdomyosarcoma of BALB-p53Neu mice [4]. It has been recently suggested that *p53* inactivation could lead to the induction of IGF2 and to the production of immature pluripotent stem cells [33]. Rhabdomyosarcoma could originate from such increased uncommitted precursors, as suggested by data on the *Ptch* rhabdomyosarcoma model [34], through the interaction with *HER-2* expression. HER-2 is best known for its involvement in breast cancer, however it has major functions in muscle cells, for example it is required for the survival of human myoblasts [35], and is frequently expressed in human rhabdomyosarcoma [11]. A further parallel with human pathology is the frequent onset of rhabdomyosarcomas in the genitourinary tract, which is the second most common site for rhabdomyosarcoma after the head and neck [36]. Finally, *P21CIP1* overexpression was observed also in human rhabdomyosarcoma [37, 38]. In conclusion, the rhabdomyosarcoma of BALB-p53Neu mice shares molecular lesions and pathological features with a subset of human embryonal rhabdomyosarcomas. This warrants the extension to human tumors of the present studies, in particular for what concerns the use of CDKN2A and p21Cip1 as potential therapeutic targets, possibly using local treatments to inhibit the pro-tumor activities of these genes without detrimental effects triggered by the inhibition of their tumor suppressor activities.

## METHODS

### Ethics statement

All the experiments were authorized by the Animal Care and Use Committee of the University of Bologna that specifically approved this study (project sent to the Italian Ministry of Health with letter n. 12511-X/10, supervisor: Prof. Carla De Giovanni), and done according to Italian and European guidelines.

### Mice

Heterozygous *p53* knockout BALB/c mice (BALB/cJ-Trp53tm1Tyj) were purchased from The Jackson Laboratory, Bar Harbor, MI. BALB/c mice transgenic for a mutant rat *neu* oncogene under control of a *MMTV-LTR* (referred to as BALB-NeuT mice) were bred in our animal facilities as described previously [39]. BALB/c *p53^+/−^*female mice were crossed with BALB-NeuT male mice then mice bearing the *p53^+/−^/neu^+/−^* genotype were selected by PCR analysis (referred to as BALB-p53Neu mice). Wild-type BALB/cAnNCrlBR mice were purchased from Charles River Italy. *Rag2^™/™^;Il2rg^™/™^* BALB/c mice, lacking T, B and NK cells were bred in our facilities from breeders kindly given by Drs. T. Nomura and M. Ito of the Central Institute for Experimental Animals (Kawasaki, Japan) [40].

### Cell lines

Cell lines RMSp53Neu-1 and RMSp53Neu-5 were established by *in vitro* culture of cells disaggregated from two primary rhabdomyosarcomas of BALB-p53Neu male mice. Cells were routinely cultured in Dulbecco's modified Eagle medium (DMEM) supplemented with 10% heat-inactivated fetal bovine serum (FBS) and were maintained at 37°C in a humidified 7% CO_2_ atmosphere. All medium constituents were purchased from Life Technologies, Milan, Italy. Cell viability was determined by the erythrosin dye-exclusion assay.

### Extraction and purification of total RNA

RNA was extracted from cultured cells, urethral tissues proximal to the urinary bladder, primary rhabdomyosarcomas or quadriceps muscles of different murine strains using TRIzol protocol (Life Technologies, Milan, Italy). Tissue samples were first disrupted using a mortar and pestle and grinded to a fine powder in liquid nitrogen, then TRIzol was added to the suspension to extract total RNA. For PCR Array analysis total RNA was purified using RNeasy Plus Micro kit (Qiagen). The protocol provided with the reagent was followed.

### PCR array analysis

The gene expression profiling analysis was performed by RT^2^ Profiler PCR Array (SABiosciences) in urethral tissues of BALB-p53Neu, BALB-NeuT and BALB/c male mice. Samples of urethral tissues were obtained from 8-week-old mice, when BALB-p53Neu mice are still free from palpable and detectable rhabdomyosarcoma. The analysis was also performed in two primary tumors. The following pathways were analyzed: Hedgehog Signaling Pathway (PAMM-078A), Mesenchymal Stem Cell Signaling Pathway (PAMM-082A), Insulin Signaling Pathway (PAMM-030A), p53 Signaling Pathway (PAMM-027A) e TGFβ Signaling Pathway (PAMM-035A).

RT^2^ First Strand Kit (SABiosciences, C-03) was used to convert 1 µg of total RNA samples into first strand cDNA. SABiosciences's RT^2^ qPCR Master Mix, based on SYBR Green detection, was used to perfom RealTime PCR according to manufacturer's instructions using a Thermal Cycler Gene Amp 5700 Detection System, Applera, Milan, Italy. A default melting curve program was used to obtain the dissociation curve for each well in the entire plate.

When Ct values were greater than 35 or genes were N/A (not detected), a value of 35 was assigned and genes were considered as not expressed. The relative quantity (Qr) of mRNA expression level of each sample was determined after normalization over the mean of five housekeeping genes, contained in PCR array plate, as endogenous reference gene using the ΔΔCt method. Then fold-changes were calculated over the mean expression of gene in all samples. To analyze expression profiles of genes, change values were then calculated between two groups (a and b):

if Qra > Qrb then change = (Qra /Qrb) - 1;
if Qra < Qrb then change = (- Qrb/Qra) + 1.

Genes having changes greater than +2 or lower than -2 were considered as up-regulated and down-regulated, respectively.

### Real-Time PCR

Gene expression was analyzed by quantitative real-time PCR using SYBR Green PCR Master Mix reagents or Taqman Universal PCR Master Mix reagents (Applera, Milan, Italy). Gene expressions evaluated with Sybr Green Real-time PCR were: *CDKN1A-p21* (forward: GGAAATCTCAGGGCCGAA; reverse: TGGGCACTTCAGGGTTTTCT); *CDKN2A-p19* (forward, GCTCTGG CTTTCGTGAACATG; reverse, CGTGAACGTTGCCCATCATC); mouse *GAPDH* (forward, GCTCACTGGCATGGCCTTC; reverse, CCTTCTTGATGTCATCATACTTGGC); *Igf-2* (forward, TTCTCATCTCTTTGGCCTTCGCCTT; reverse, ATATTGGAAGAACTTGCCCACGGGG) [41]; *TrP53* (forward, CCCGAGTATCTGGAAGACAG; reverse, ATAGGTCG GC GGTTCAT). Gene expressions evaluated with TaqMan Real-time PCR were: rat *HER-2* (forward, GCAACTTGGAGCTTACCTACG; reverse, GCATGA-GCATGTAACCCTGA; probe, CCAGCCTCTCATTCC); rodent *GAPDH* (Applied Biosystem).

### Gene silencing with siRNA

Murine rhabdomyosarcoma cell lines were seeded in 6-well Multiwell plates (Falcon, Becton Dickinson, Franklin Lakes, USA) in DMEM +10% FBS without antibiotics. The doses of seeding were: RMSp53Neu-1, 1×10^4^ cells/cm^2^, RMSp53Neu-5, 1.5×10^4^ cells/cm^2^. After 24 h cells were transfected with siRNA following protocol suggested by the manufacturers.

*p21Cip1* mouse siRNA (catalog number 1024837, *CDKN1A* reference sequence NM_007669 [42]) and Allstars Negative siRNA (hereafter referred to as “Non-targeting 1”) labeled with Alexa Fluor 488 (catalog number 1027284) were synthesized and purchased from Qiagen, Milan. Mouse *CDKN2A* ON-TARGETplus SMARTpool siRNA (catalog number L-043107-00, reference sequence NM_009877) and Control Non-targeting ON-TARGETplus Pool siRNA (catalog number D-001810-10, hereafter referred to as “Non-targeting 2”) were purchased from DHARMACON, Chicago. siRNA were used at a concentration of 40 nM using Lipofectamine RNAiMax (Life Technologies) as transfection agent (0.02%).

### Western Blot

Cells were lysed with 50 mM Tris-HCl (pH 7.5), 1 mM EDTA, 1% Igepal, 0.5% sodium deoxycholate, 0.1% SDS, 10% glycerol, 150 mM NaCl plus phosphatase and protease inhibitors (all reagents were purchased from Sigma, Milan, Italy) for 30 min on ice. Nuclei were removed by centrifiugation at 12,000 x g at 4°C for 15 min and protein concentration in the supernatants was determined by DC Protein Assay (Bio-Rad, Milan, Italy) using BSA as standard. Proteins were separated on a 12% polyacrylamide gel (30 μg for p19Arf and p21Cip1, 10 μg for actin evaluation) then transferred to polyvinylidene difluoride membranes (Bio-Rad). After blocking with PBS containing 0.1% tween 20 plus 5% non-fat dry milk for two hours at room temperature, membranes were incubated overnight at 4°C with primary antibodies diluted in blocking buffer. Anti-p19Arf rat monoclonal antibody (5-C3-1) 0.7 μg/ml and anti-p21 mouse monoclonal antibody (F-5) 0.7 μg/ml (both purchased from Santa Cruz Biotechnology, Santa Cruz, CA) and anti-actin rabbit antibody 1 μg/ml (purchased from Sigma, Milan, Italy) were used as primary antibodies. After incubation with the respective horseradish peroxidase-labeled secondary antibodies (Santa Cruz Biotechnology), protein presence was revealed by chemiluminescent reaction (LiteAblotplus, Euroclone, Milan, Italy).

### Effect of gene silencing on cell growth

Cell yield was determined after 24, 48 and 72 h of siRNA treatment. To evaluate the effects on cloning efficiency and on motility cells were then harvested and reseeded in the absence of siRNA. To measure cloning efficiency RMSp53Neu-1 (100 cells) and RMSp53Neu-5 (200 cells) cells were seeded in 60-mm tissue culture Petri dishes in DMEM + 10% FBS. After 8-11 days, colonies were fixed in ethanol, stained with Giemsa and counted with an inverted microscope at low magnification (25×). Cell migration assay was performed using Transwell chambers (Costar) with 8-µm pore size, polyvinylpyrrolidone-free polycarbonate filters (Corning, Cambridge, MA). Serum-free DMEM was put in the lower compartment. 1×10^5^ murine rhabdomyosarcoma cells were seeded in 100 µl serum-free DMEM in the upper compartment of the Transwell chambers and incubated for 18 hours. Cells which migrated through the filter to reach the lower chamber were counted at the inverted microscope. Anchorage-independent growth was determined by suspending cells in DMEM + 10% FBS containing 0.33% agar. Cell suspensions were seeded at 6250, 12000 and 25000 cells/Petri dish on a 5-ml base of 0.5% agar in 60-mm Petri dishes. Colony growth was monitored weekly and determined by counting at low magnification (25×) 14 days after seeding.

### Effect of gene silencing on metastasis

The effect p21Cip1 or CDKN2A silencing on metastatic capacity of murine rhabdomyosarcoma cells were evaluated in BALB-NeuT male mice. These mice received intravenous (i.v) injections of 0.4 ml PBS containing 2×10^5^ RMSp53Neu-1 viable cells or 3×10^5^ RMSp53Neu-5 viable cells pretreated with siRNAs for 48-h. To increase detection of liver metastasization ability, *Rag2^–/–^;Il2rg^–/–^* male mice (6-20 weeks-old), were also used for some experiments with RMSp53Neu-1 cells treated with siRNA anti-*CDKN2A*. These immunodeficient mice had received i.v injection of 5×10^4^ RMSp53Neu-1 viable cells in 0.4 ml PBS. Mice were sacrificed after 28 days and were subjected to an accurate necropsy; lungs were stained with black India ink to better outline metastases and fixed in Fekete's solution. Lung and liver metastases were counted using a dissection microscope.

## Supplemental Figure


