# Supplementary material for: Tumor suppressor genes promote rhabdomyosarcoma progression in p53 heterozygous, HER-2/neu transgenic mice
Source: Oncotarget. 2013 Aug 6;5(1):108–19. doi: 10.18632/oncotarget.1171 (PMC3960193; doi:10.18632/oncotarget.1171)
Supplement: Supplementary file 1 [file oncotarget-05-0108-s001.pdf]

Tumor suppressor genes promote rhabdomyosarcoma progression in p53 heterozygous, HER-2/neu transgenic mice - Ianzano et al

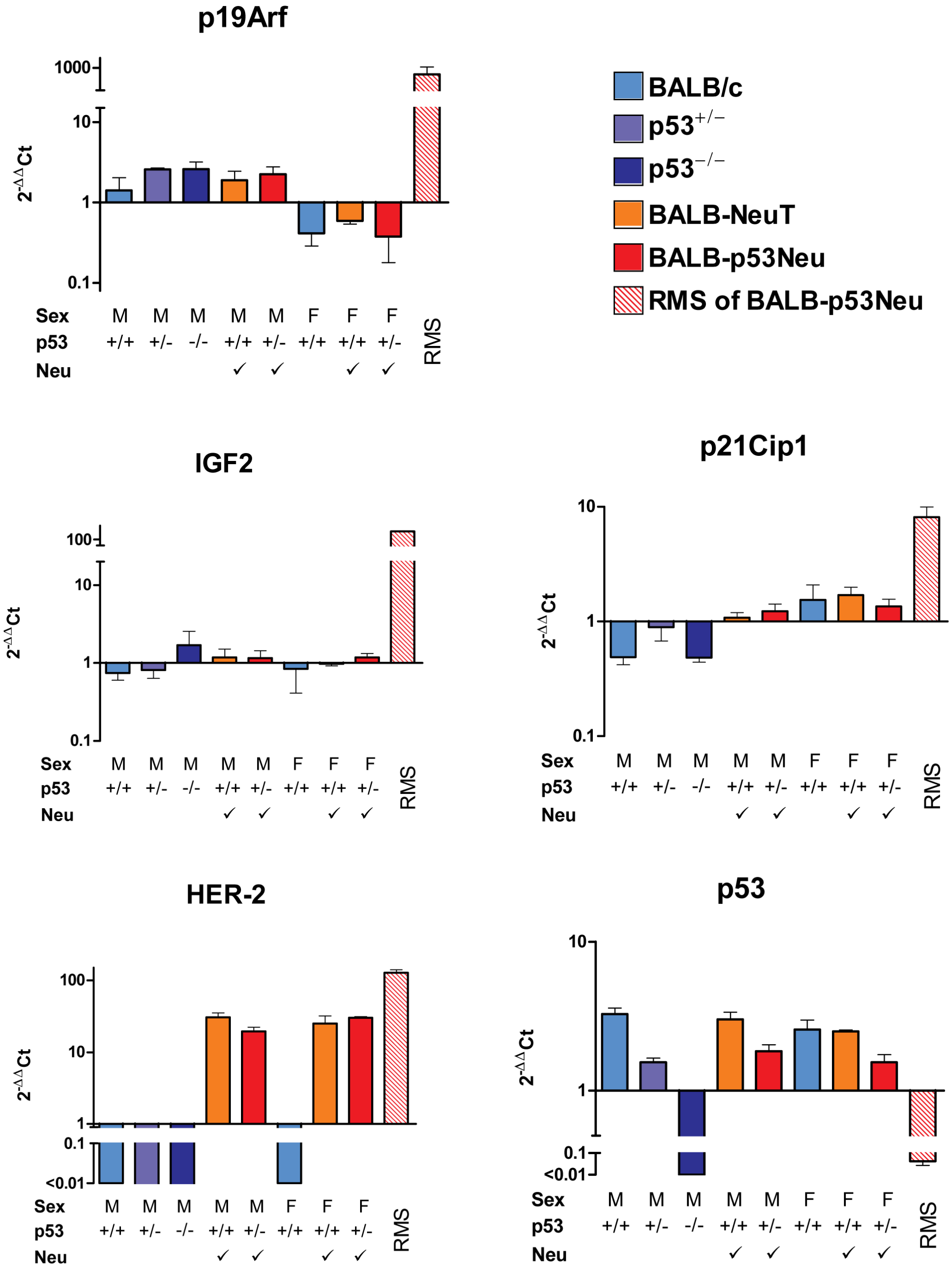

Supplementary Figure 1: Gene expression in quadriceps muscles of 8-week-old mice and in rhabdomyosarcomas (RMS) of BALB-p53Neu mice. Each panel represents the expression of the indicated gene in samples obtained from mice differing in sex (M/F), *p53* status (*p53*$^{+/+}$, *p53*$^{+/-}$ or *p53*$^{-/-}$) and presence of a HER-2/neu transgene (✓), as indicated under each bar. Each bar in a panel represents mean ± SEM gene expression level relative to the average of all samples (excluding RMS). Mean threshold cycles were: *GAPDH*, 17.5; *p19Arf*, 35.2; *Igf2*, 25.6; *p21Cip1*, 26.1; *HER-2/neu*, 33.2; *p53*, 26.9. The number of mice in each group was: BALB/c male mice (M +/+), 3; p53 heterozygous BALB/c males (M +/−), 3; p53 knockout males (M −/−), 2; male BALB-NeuT (M +/+ ✓), 3; male BALB-p53Neu (M +/− ✓): 4; female BALB/c (F +/+), 4; female BALB-NeuT (F +/+ ✓), 3; female BALB-p53Neu (F +/− ✓), 3; rhabdomyosarcomas (RMS) of male BALB-p53Neu (same samples as in Figure 1), 2.
